# Attitudes, Knowledge, and Perceived Barriers Towards Cancer Pain Management Among Healthcare Professionals in Libya: a National Multicenter Survey

**DOI:** 10.1007/s13187-022-02185-5

**Published:** 2022-06-02

**Authors:** Salim M. Makhlouf, Shenaz Ahmed, Matthew Mulvey, Michael I. Bennett

**Affiliations:** Academic Unit of Palliative Care, Leeds Institute of Health Sciences, School of Medicine, University of Leeds, Level 10 Worsley Building, Clarendon Way, Leeds, LS2 9NL UK

**Keywords:** Cancer pain management, Healthcare professionals, Knowledge, Attitudes, Perceived barriers, Libya

## Abstract

Cancer pain presents in approximately 66% of patients in advanced stages. Although several guidelines and pharmacological options are available for cancer pain management (CPM), assessment and treatment of cancer pain remain inadequate globally, particularly in developing countries. Lack of knowledge and negative attitudes towards CPM among healthcare professionals (HCPs) are important barriers to CPM. This survey aimed to evaluate nurses’ and physicians’ knowledge, attitudes, and potential barriers regarding CPM in Libya. This cross-sectional survey involved a convenience sample of 152 oncology nurses and physicians working in six oncology settings in Libya. The response rate was 76%. The Barriers Questionnaire II (BQ-II) was used for data collection (higher scores signify greater attitudinal barriers and poorer knowledge). Data analysis was carried out using Statistical Package for Social Sciences (SPSS), version 26 software. An independent *t*-test (unadjusted estimate) indicated that Libyan nurses showed higher mean barrier scores (mean = 3.8, *SD* = 0.7) to CPM than physicians (mean = 2.9, *SD* = 0.8), *p* < 0.001. The six most common differences in attitudinal barriers between nurses and physicians were “opioid side effects,” “poor tolerance,” “strong patient endures pain,” “distract the physician,” “drug addiction,” and “opioids impair immune function,” *p* < 0.001. Multiple regression results (adjusted estimate) indicated that nurses had more barrier scores to CPM than physicians (B =  − 0.530, *p* < 0.05), and participants with higher educational levels were associated with lower barrier scores to CPM (B =  − 0.641, *p* < 0.05). Our results suggest that Libyan oncology HCPs hold perceived barriers, lack of knowledge, and negative attitudes towards CPM. Professional education and training in CPM, addressing phobia and myths on opioid usage, and the benefits and complications of using opioids are likely to result in reduced barriers to CPM in Libya.

**Supplementary Information:**

The online version contains supplementary material available at 10.1007/s13187-022-02185-5.

## Introduction

Cancer pain is a major international health problem, as it is often undertreated in many cases [1]. Such pain can result from the disease itself, metastases associated with a tumor, or nerve damage, which might be caused by cancer treatments [2]. Pain associated with cancer negatively affects patients’ and their caregivers’ quality of life (QoL) [1]. A meta-analysis conducted in the USA with 169 studies on pain in patients with cancer reported that approximately 55% of cancer patients in active anti-cancer treatment and more than 65% of patients in advanced, metastatic, and terminal stages experienced cancer pain [1].

Although various guidelines and pharmacological options are available to manage pain in patients with cancer, assessment and management of cancer pain remain inadequate worldwide [3], particularly in developing countries [4]. A survey conducted in Lebanon that included 400 cancer patients found that more than 37% of cancer patients suffer from cancer pain, and inadequate CPM was reported to be about 46% among all cancer patients [4]. Some studies from the same regions suggested that cancer pain is unrelieved in many cases due to limited access or legal restrictions to opioids and rejection of the uses of opioids by HCPs [5]. However, studies conducted in the USA found that many cancer patients were experiencing unrelieved cancer pain, although they had increasingly been prescribed opioids for their pain [6]. Thus, accessing opioids alone is unlikely to relieve cancer pain [7], as other factors can prevent effective CPM. Such factors include physiological effects, e.g., fear of drug tolerance and side effects; fatalistic beliefs; communication, e.g., a strong patient does not complain about pain; and harmful effects, e.g., addiction to opioids [7].

Lack of knowledge and poor attitudes towards CPM among HCPs were reported in developing countries and worldwide [3] as the most common barriers to CPM. A recent systematic review reported that HCPs’ barriers toward CPM related to a lack of knowledge and negative attitudes leading to unalleviated pain in most cancer patients [3].

Similar to some Arab countries in the Middle East and North Africa (MENA) region, Libya lacks certain healthcare services, such as pain management and palliative care [5]. Although the absence of these services was reported as a system-related barrier to effective CPM, many researchers indicated that HCPs, who have experience in pain clinics and palliative care settings, showed better attitudes towards opioids and knowledge about CPM than those who did not [8]. Furthermore, several studies conducted internationally, which assessed HCPs’ attitudes, knowledge, and perceived barriers towards CPM, found that one of many common barriers towards CPM was the HCPs’ lack of knowledge and training in CPM and negative attitudes towards CPM [3]. Moreover, the CPM situation among Libyan HCPs has not been previously assessed, despite the poor QoL, which has been found among cancer patients in Libya [9]. Therefore, this study evaluates nurses’ and physicians’ knowledge, attitudes, and potential barriers regarding cancer pain and its management in Libya.

## Methods

### Participants and Settings

A cross-sectional survey was carried out with a convenience sample of 152 eligible participants (oncology nurses and physicians) working in several oncology settings (*n* = 6), which were located in three different regions of Libya (Eastern, North-western, and Western). These are Tobruk Medical Centre (TMC), Benghazi Medical Centre (BMC), National Cancer Centre Benghazi (NCCB), National Cancer Institute of Misratah (NCIM), National Oncology Institute of Sabratha (NOIS), and Tripoli Medical Centre (TMC). After receiving ethical approval from the School of Medicine Research Ethics Committee, University of Leeds, the UK, and relevant settings in Libya, the targeted participants were recruited by survey coordinators (oncology physicians) between November 2020 and April 2021. The response rate was 76%. To minimize the bias, all participants were recruited through survey coordinators, oncology HCPs (not senior or manager staff) at each national oncology setting in Libya. The first author (SM) sent the questionnaires and information sheets via Dropbox to the survey coordinators at each national oncology setting in Libya. Each survey coordinator printed and distributed the questionnaires with an information sheet to all potential participants, as instructed to minimize the potential bias. After the questionnaires were completed, the participants put the completed questionnaires in the secure boxes themselves. Then the survey coordinators scanned and uploaded all completed questionnaires into Dropbox and sent them to the first author. After all questionnaires were sent, each coordinator was instructed to safely delete electronic copies and shred all hard copies.

### Survey Instrument

The Barriers Questionnaire (B.Q.) was developed by Ward et al. [10], and it was revised and renamed the Barriers Questionnaire II (BQ-II) [7], which was used for data collection in this study. Permission was obtained to use the BQ-II in this survey. The BQ-II consists of 27 multiple-choice questions (self-report questionnaire) divided into four subscales: physiological effects, fatalism, communication, and harmful effects. The survey items are measured on a 6-points Likert scale, which shows how much the participant agrees with the target statement. For instance, “0” means “do not agree at all,” and “5” means that the participant is “agreed very much” [10]. This self-report questionnaire assesses concerns about cancer pain and using pain medication for CPM [10]. It also evaluates the attitudinal barriers toward CPM [7]. Mean scores for the BQ-II overall scale and subscales are used as dependent (outcome) variables for analyses, with higher scores (rating 3 or above: > 50%) indicating greater attitudinal barriers and poorer knowledge about CPM. Items 1, 8, and 24 in subscale (fatalism) were reverse scored before starting data analysis. Minor changes were made, such as the word patient/s was used instead of the phrase “you and I” to fit this study’s purpose. Answers “mostly agree” and “agree” were merged into the category “agree very much,” indicating barriers toward CPM.

The BQ-II has been shown to be a reliable and valid instrument to measure patient, family caregiver, and HCPs related barriers to CPM in several studies [7, 11–14]. Based on the findings from Gunnarsdottir et al. [13], there is the initial evidence of both the reliability and validity of the BQ-II. It is a well-known questionnaire, as it has been validated and used in different studies [7, 11, 14] in different languages, including Arabic [11, 12, 14]. According to Al Khalaileh and Al Qadire [11], the BQ-II was “translated into Arabic and verified using the back-translation approach, and a linguistic expert was consulted to ensure that the translation was adequate.” (p.2). This approach is well-known as it is usually used when translating such survey instruments [15]. Permission to use an Arabic version of the BQ-II was obtained for the current study. An Arabic version of the BQ-II has been validated and used in previous studies [11, 12, 14]. It has been recommended to pilot test an instrument to ensure general readability before using it in a study that involves a new target population [16]. Therefore, an Arabic version of the BQ-II was pilot tested with eight participants (who were included in the study) before distributing the questionnaire to all study participants to ensure readability. The psychometric properties for the Arabic version of BQ-II are reported here using response data from the entire final sample size; *n* = 152 participants, including test–retest reliability (*r* > 0.80) and internal consistency reliability (the Cronbach’s Alpha) for the overall BQ-II scales for HCPs was excellent (α = 0.90) and Alpha for the three factors ranging from 0.74 to 0.85.

### Statistical Analysis

Descriptive statistics were used to analyze demographic information, including age, gender, marital status, profession (nurses vs. physicians), educational background (high-school vs. undergraduate vs. postgraduate degrees), training, years of work experience, and using WHO for CPM. Categorical data are summarized as numbers (proportion), and continuous data are summarized using means (standard deviation) and range. Inferential analyses included an independent *t*-test and multiple linear regression analysis to analyze the relationship between dependent (outcome) and independent (cause) variables [16]. The statistical significance level was set at a 2-sided *p* < 0.05. Data coding and analysis were carried out using Statistical Package for Social Sciences (SPSS), version 26 software. Since this is an exploratory study, we do not intend for readers to treat the results as definitive, and, as such, we do not make corrections/adjustments for multiple testing.

## Results

### Sample Characteristic

Two hundred Libyan oncology nurses and physicians consented to participate in this study. For this cross-sectional survey, 185 (93%) HCPs responded. Of those 185, 160 (87%) participants returned the questionnaires (via secured boxes) to the survey coordinators. Eight of the returned questionnaires were excluded as many answers were missing. Thus, 152 (76%) valid questionnaires were eligible for use in this study for statistical analyses. According to Guadagnoli and Velicer [17], a sample size between 100 and 150 is recommended, especially when the internal consistency for an instrument is expected to be at α = 0.60 and above. In the current study, the internal consistency reliability (ICR) was calculated for the entire sample size (*n* = 152) participants. The ICR for the overall BQ-II scales in this study was higher (α = 0.90) than recommended, indicating an excellent internal consistency for the overall BQ-II scales [18].

The mean age for all respondents was 36.29 years (*SD* = 7.5), ranging between 20 and 64. Participants mostly were females (65.1%), and there were more physicians (62.5%) than nurses (37.5%). There were more married participants (51.3%) than single (40.1%). Physicians were more likely to have attained university education than nurses (undergraduate degree: 61.1% versus 14.0% and postgraduate: 38.9% versus 1.8%). The majority of nurses (84.2%) only held high-school-level qualifications (equivalent to UK A levels). A few participants (12.5%) had completed training on CPM. More participants (84.9%) had long-term (greater than 1 year) work experience in cancer settings than those who had short-term (less than 1 year) work experience (15.1%). Participant characteristics are presented in Table 1.

**Table 1:** Participants’ demographic characteristics

HCPs (oncology nurses and physicians) (*n* = 152)	Respondents
Profession; *n* (%): Nurses Physicians	57 (37.5) 95 (62.5)
Gender; *n* (%): Male Female	53 (34.9) 99 (65.1)
Age (years): Mean (SD) Range	36.29 (7.5) 20–64
Marital status; *n* (%): Single Married Divorced Widowed Missing	61 (40.1) 78 (51.3) 6 (3.9) 1 (0.7) 6 (3.95)
Education; *n* (%): High-school degree Undergraduate degree Postgraduate degree	48 (31.6) 66 (43.4) 38 (25.0)
Annual salary Mean (SD) Range (£/year) Missing	0.80 (0.67) < 8000–14,400 66 (43.42)
Training in CPM; *n* (%): Yes No	19 (12.5) 133 (87.5)
Work experience; *n* (%): < 1 year > 1 year	23 (15.1) 129 (84.9)
WHO and NICE for CPM; *n* (%): Yes No	65 (42.8) 87 (57.2)
Medication for CPM; *n* (%): Non-opioids Weak opioids Non-opioids and weak opioids Non-opioids, weak opioids, and strong opioids Missing	12 (8.2) 5 (3.5) 42 (28.6) 88 (59.9) 5 (3.5)

*CPM*, cancer pain management; *HCP*, healthcare professionals; *n*, number; *SD*, standard deviation; %, percentage; £, pound sterling; < , less than; > , greater than

### Barriers to Effective CPM Among HCPs

#### Barriers Related to Poor Attitudes Towards CPM

Before the multiple linear regression test was run, bivariate analysis such as an independent *t*-test was conducted to compare the outcome variables (an overall 27 items of BQ-II and its subscales) between nurses and physicians (unadjusted estimate) regarding their attitudinal barriers toward CPM. The overall mean on the BQ-II item scores for nurses and physicians was 3.3 (*SD* = 0.8), indicating high barriers to CPM. The 95% confidence interval (*CI*) for the mean ranged from 0.64 to 1.12. The equality of variances (homogeneity of variances) was assumed for all analyses using Leven’s test (*p* > 0.05). The result showed that nurses showed higher mean barrier scores (mean = 3.8, *SD* = 0.7) to CPM than physicians (mean = 2.9, *SD* = 0.8), *p* < 0.001. The results showed attitudinal variations in CPM between nurses and physicians on the most subscale items (see Table 2). Only five items did not show differences between nurses and physicians. No attitudinal difference between the two groups (nurses and physicians) was seen for one item on the “physiological effects” subscale “If the patient took pain medicine when he/she had mild pain, such medication might not be effective as well if his/her pain became severe,” *p* > 0.05, one item on the “harmful effects” subscale “Many patients with cancer can be addicted to pain medication,” *p* > 0.05, and three items on the “fatalism” subscale “Cancer pain can be relived, pain medicine can effectively control cancer pain, and medication can relieve pain related to cancer,” *p* > 0.05.

**Table 2:** Perceived barriers to CPM on the BQ-II by Libyan HCPs (*n* = 152), using an independent *t*-test (unadjusted estimate)

Items in the questionnaire	Mean scores (SD)		95% *CI*	*p*-value
	Nurses	Physicians		
1. Drowsiness from pain medicine is difficult to control	3.1 (1.7)	2.4 (1.5)	0.175, 1.221	0.009**
2. Confusion from pain medicine cannot be controlled	3.0 (1.6)	2.2 (1.4)	0.306, 1.273	0.002**
3. When the patient uses pain medicine, his/her body becomes used to its effects, and, pretty soon, it will not work anymore	4.0 (1.0)	3.5 (1.4)	0.137, 0.901	0.008**
4. Using pain medicine blocks the patient’s ability to know if he/she has any new pain	3.5 (1.5)	3.0 (1.7)	0.242, 1.316	0.005**
5. Nausea from pain medicine cannot be relieved	2.5 (2.0)	1.3 (1.3)	0.631, 1.783	0.000***
6. Pain medicine makes patients say or do embarrassing things	2.5 (1.9)	1.6 (1.4)	0.289, 1.452	0.004**
7. If a patient takes pain medicine when he/she has some pain, then it might not work as well if the pain becomes worse	3.5 (1.5)	3.2 (1.5)	− 0.233, 0.766	0.293
8. Pain medicine can keep patients from knowing what is going on in their bodies	3.3 (1.6)	2.2 (1.7)	0.473, 1.569	0.000***
9. Constipation from pain medicine cannot be relieved	2.5 (1.7)	1.4 (1.4)	0.525, 1.587	0.000***
10. It is easier for a patient to put up with pain than the side effects that come from pain medicine	3.3 (1.7)	2.1 (1.8)	0.563, 1.697	0.000***
11. If the patient uses pain medicine now, it will not work as well if he/she needs it later	3.4 (1.6)	2.6 (1.7)	0.243, 1.357	0.005**
12. Pain medicine can mask changes in the patient’s health	3.3 (1.7)	2.6 (1.6)	0.217, 1.298	0.006**
13. Cancer pain can be relived	1.3 (1.4)	1.2 (1.16)	− 0.325, 0.549	0.611
14. Pain medicine can effectively control cancer pain	1.5 (1.3)	1.5 (1.4)	− 0.416, 0.486	0.878
15. Medicine can relieve cancer pain	1.2 (1.4)	1.1 (1.2)	− 0.385, 0.462	0.857
16. It is important for the patient to be strong by not talking about his/her pain	3.3 (1.7)	1.8 (1.9)	0.809, 1.998	0.000***
17. It is important for the doctor to focus on curing illness and not waste time controlling pain	3.8 (1.7)	1.5 (2.0)	1.627, 2.829	0.000***
18. If doctors have to deal with the pain, they will not concentrate on curing the disease	2.1 (1.9)	1.2 (1.7)	0.358, 1.586	0.002**
19. Doctors might find it annoying to be told about the pain	2.2 (2.0)	1.0 (1.5)	0.615, 1869	0.000***
20. Reports of pain could distract a doctor from curing cancer	2.1 (2.0)	1.1 (1.6)	0.378, 1.594	0.002**
21. If the patient talks about pain, people will think he/she is a complainer	2.9 (2.0)	2.0 (2.0)	0.438, 1.717	0.001**
22. There is a danger of patients becoming addicted to pain medicine	4.0 (1.4)	3.2 (1.5)	0.306, 1.273	0.002**
23. Pain medicine weakens the immune system	3.0 (1.8)	1.9 (1.7)	0.580, 1.736	0.000***
24. Many people with cancer get addicted to pain medicine	4.0 (1.5)	3.4 (1.5)	− 0.072, 0.914	0.094
25. Using pain medicine can harm a patient’s immune system	3.5 (1.7)	1.7 (1.7)	1.245, 2.362	0.000***
26. Pain medicine can hurt a patient’s immune system	3.4 (1.7)	1.7 (1.7)	1.202, 2.314	0.000***
27. Pain medicine is very addictive	3.8 (1.6)	3.0 (1.5)	0.277, 1.274	0.003**
Overall mean scores for the BQ-II	3.8 (0.7)	2.9 (0.8)	0.64, 1.12	0.000***

*SD*, standard deviation; %, percentage; *CI*, confidence interval; *p*-*value*, the probability; **p < 0.05; ***p < 0.001

The major differences of attitudinal barriers towards CPM between nurses and physicians on the “physiological effects subscale” were concerned about “side effects” (mean = 3.3, *SD* = 0.9 for nurses and mean = 2.4, *SD* = 0.7 for physicians) and concerned “poor tolerance” (mean = 3.9, *SD* = 0.7 and mean = 3.2, *SD* = 0.9), respectively, *p* < 0.001. Whereas on “communication” were “strong patient does not complain about pain” (mean = 2.7, *SD* = 0.9, for nurses and mean = 1.9, *SD* = 1.0, for physicians) and “pain can distract the physician from treating cancer” (mean = 2.8, *SD* = 1.2 and mean = 1.8, SD = 1.0), respectively, *p* < 0.001. While on “harmful effects” were “fear of drug addiction,” mean = 3.64 and *SD* = 0.82 for nurses compared to mean = 3.15 and *SD* = 0.97 for physicians, and fear of “opioids impair patient’s immune function” (mean = 3.23, *SD* = 1.12 and mean = 2.05, *SD* = 1.20), respectively, *p* < 0.001. However, there was no difference between nurses and physicians on “fatalistic beliefs,” *p* > 0.005. Nurses had higher attitudinal barrier scores towards CPM than physicians, *p* < 0.001. Table 2 shows all different respondents’ perspectives on barriers to CPM between nurses and physicians.

#### Barriers Related to Lack of Knowledge About CPM

Libyan nurses had higher barrier scores (mean = 3.8, *SD* = 0.7) to CPM than physicians (mean = 2.9, *SD* = 0.8), *p* < 0.001. The most common responses on the BQ-II subscales (rating 3 or above: > 50%; indicating poorer knowledge of CPM) between nurses and physicians were 70% (*n* = 40/57) for nurses and 43% (*n* = 41/95) for physicians, for the statement “Using pain medicine can block the patient from knowing what is going on in his/her body.” Furthermore, 53% (*n* = 30/57) of nurses thought “drug side effects, such as ‘constipation’ is difficult to relieve” compared with 15% (*n* = 24/95) of physicians. Moreover, 72% (*n* = 41/57) of nurses and 34% (*n* = 34/95) of physicians believed that “a strong patient does not complain about pain.” In addition, 81% (*n* = 46/57) of nurses compared to 31% (*n* = 29/95) of physicians completely agreed that pain could “distract the doctor” for the statement “doctors should focus on curing cancer and not wasting their time by controlling pain.” Besides, 84% (*n* = 48/57) of nurses expressed deep concern about “opioids addiction” compared to 69% (*n* = 66/95) of physicians. Additionally, the harmful effects of “opioids can impair a patient's immune system” also caused concern among nurses 70% (*n* = 40/57) and physicians 27% (*n* = 26/95). This result indicates that Libyan oncology physicians have a higher understanding of opioids and CPM than nurses. However, differences reported above are based on an independent *t*-test (unadjusted estimate) which cannot account for multiple relationship. Thus, a multiple linear regression analysis was needed to investigate whether the Libyan HCPs’ demographic variables (age, gender, marital status, profession, educational level, training, work experience, or using WHO for CPM) were significantly associated with participants’ mean overall BQ-II and its subscale scores (adjusted estimate).

### Multiple Regression for the Mean Overall BQ-II

Multiple linear regression analyses were performed to determine whether the Libyan HCPs’ demographic variables (e.g., age, marital status, gender, profession, educational level, training, work experience, or using WHO for CPM) were related to their mean overall BQ-II scores (adjusted estimate). All of the assumptions were met. The overall model explains a 33.1% variation of mean overall BQ-II scores, and it is significantly useful in explaining mean overall BQ-II scores, *F* (11, 134) = 6.014, *p* < 0.05. The results showed that the profession (nurses vs. physicians) contributed significantly to the model, as the mean barriers to CPM (the overall BQ-II scores) decreased by − 0.530, which was found to be a significant change, *t* (134) =  − 1.998, *p* < 0.05. This indicates that profession was significantly associated with participants’ mean overall BQ-II scores; the physicians had lower barrier scores to CPM compared to the nurses. The results also showed that with a one-unit increase in educational level, the mean barriers to CPM (the overall BQ-II scores) decreased by − 0.641, which was found to be a significant change, *t* (134) =  − 2.121, *p* < 0.05. This result shows that participants with postgraduate degrees showed lower barrier scores to CPM (B =  − 0.641) than those with a high-school degree and undergraduate degree (B =  − 0.082). However, age, gender, marital status (single vs. married vs. divorced), training, work experience, using WHO for CPM did not significantly associate with the mean overall BQ-II scores. This result indicates that age, gender, marital status, training, work experience, and WHO for CPM did not contribute to the multiple regression model (see Table 3).

**Table 3:** Summary of multiple linear regression findings between mean overall BQ-II and the socio-demographic factors (adjusted estimate)

Variables	Levels	Beta coefficients	*R*^2^	Coefficient (95% *CI*)	*t*-value	*p*-value
Constant		4.547	0.331	2.610, 6.483	4.644	0.000***
Age		0.040	0.331	− 0.239, 0.319	0.283	0.778
Gender	Ref = Male	-	-	-	-	-
	Female	− 0.082	0.331	− 0.343, 0.178	− 0.624	0.534
Marital status	Ref = Widowed	-	-	-	-	-
	Single	0.119	0.331	− 1.351, 1.590	0.161	0.873
	Married	0.169	0.331	− 1.295, 1.633	0.228	0.820
	Divorced	− 0.572	0.331	− 2.134, 0.990	− 0.724	0.470
Profession	Ref = Nurses	-	-	-	-	-
	Physicians	− 0.530	0.331	− 1.054, − 0.005	− 1.998	0.048**
Education	Ref = Undergraduate	-	-	-	-	-
	High-school	− 0.208	0.331	− 0.753, − 0.336	− 0.757	0.450
	Postgraduate	− 0.641	0.331	− 1.239, − 0.043	− 2.121	0.036**
Training		− 0.261	0.331	− 0.634, 0.113	− 1.380	0.170
Work experience		− 0.106	0.331	− 0.451, 0.240	− 0.604	0.547
WHO for CPM		0.088	0.331	− 0.175, 0.352	0.664	0.508

*p-value*, the probability; *CI*, confidence interval; *Overall BQ-II scores*, the 27 items on Barriers Questionnaire II; *R*^*2*^, coefficient of determination; %, percentage; *n*, number; **p < 0.05; ***p < 0.001

### Multiple Regression for the Mean BQ-II Subscales

Multiple linear regression analysis also was fitted to determine whether the nurses’ and physicians’ demographic variables (e.g., age, gender, marital status, profession, educational level, training, work experience, and WHO for CPM) were related to their mean BQ-II subscale physiological effect scores (adjusted estimate). All of the assumptions were met. The overall model explains a 26.7% variation of mean BQ-II subscale physiological effect scores, and it is significantly useful in explaining mean BQ-II physiological effect scores, *F* (11, 134) = 4.427, *p* < 0.05. Multiple linear regression indicated that the profession (nurses vs. physicians) contributed significantly to the model, as the mean barriers to CPM (the BQ-II subscale physiological effect scores) decreased by − 0.615, which was found to be a significant change, *t* (134) =  − 2.224, *p* < 0.05. This indicates that profession was significantly associated with participants’ mean BQ-II subscale physiological effect scores; the physicians had lower CPM barrier scores than the nurses (B =  − 0.615). Nevertheless, there is no longer a mean grade difference between age: *t* (134) = 1.018, *p* > 0.05; gender: *t* (134) = 0.229, *p* > 0.05; marital status (single vs. married vs. divorced): *t* (134) = 0.366, *p* > 0.05, *t* (134) = 0.375, *p* > 0.05, *t* (134) =  − 0.484, *p* > 0.05; educational levels (high-school vs. undergraduate vs. postgraduate degrees): *t* (134) = 0.065, *p* > 0.05, *t* (134) =  − 1.025, *p* > 0.05; training: *t* (134) =  − 1.940, *p* > 0.05; work experience: *t* (134) =  − 0.420, *p* > 0.0.5; and WHO for CPM: *t* (134) = 1.131, *p* > 0.0.5. The result shows that age, gender, marital status, educational level, training, work experience, and WHO for CPM did not contribute to the multiple regression model.

Multiple linear regression analyses also were performed to determine whether the HCPs’ demographic variables (age, gender, marital status, profession, educational level, training, work experience, WHO for CPM) were related to their mean BQ-II subscales “fatalism”, “communication,” or “harmful effects” scores (adjusted estimate). All of the assumptions were met. The results showed that for “fatalism,” only marital status (single vs. married vs. divorced vs. widowed) was significantly associated with the mean BQ-II subscale fatalism scores (B =  − 3.011, *p* < 0.05; B =  − 3.007, *p* < 0.05; B =  − 2.770, *p* < 0.05). This result means that those who were single (B =  − 3,011), married (B =  − 3.007), and divorced (B =  − 2.770) experienced more mean barrier scores on the subscale “fatalism” BQ-II than those who were widowed. For “communication” however, only educational level (high-school vs. undergraduate vs. postgraduate degrees) contributed significantly to the model (B =  − 1.692, *p* < 0.001 and B =  − 2.072, *p* < 0.001), respectively. This result shows that participants with high-school and undergraduate degrees had more barrier scores to CPM (B =  − 1.692) than those with a postgraduate degree (B =  − 2.072). However, for “harmful effects,” only profession (nurses vs. physicians) and education (high-school vs. undergraduate vs. postgraduate degrees) contributed significantly to the model (B =  − 1.614, *p* < 0.001 and B = 1.123, *p* < 0.05, respectively). This result illustrates that nurses had more barrier scores to CPM than physicians (B =  − 1.614), and the participants with high-school and undergraduate degrees had more barrier scores to CPM (B = 1.123) than those with a postgraduate degree (B = 0.363).

## Discussion

This study examined the nurses’ and physicians’ knowledge, attitudes, and potential barriers regarding cancer pain and its management in Libya. This is the first survey to evaluate knowledge, attitudes, and potential barriers regarding CPM among Libyan nurses and physicians, to the best of our knowledge. Similar to previous studies [3, 19], the results in this study showed that Libyan oncology nurses had a high level of barriers to CPM than physicians. However, both nurses and physicians in the current study had a higher barrier score on the overall and subscale BQ-II items compared to previous studies [11, 12].

In this study, the most significant differences between nurses and physicians were related to the barrier items, including those concerning side effects of opioids, poor tolerance, and drug addiction. This finding is similar to previous studies [19]. HCPs who believe that strong opioids for CPM can lead to poor tolerance, drug addiction, and side effects are more likely to undertreat patients with cancer pain. A study found that about 73% of physicians hesitated to increase opioid dosage and frequency for CPM due to the unwarranted fear of drug tolerance and addiction [20].

This study also found that Libyan nurses had higher barrier scores on the BQ-II subscales (physiological and harmful effects) than physicians, indicating a lack of adequate knowledge about CPM among Libyan oncology nurses. Compared to previously published studies [11, 12], this study found higher barrier scores on the psychological effects and harmful effects of BQ-II subscales. Moreover, our survey found higher barrier scores on the communication of BQ-II subscales compared to previous studies [11, 12]. These differences in barrier scores indicate that oncology HCPs in Libya showed a higher lack of knowledge about CPM than HCPs in other countries. A study suggested that HCPs dealing with cancer patients should have adequate knowledge about cancer pain and its management to improve CPM [21].

As shown by other studies [19, 22], Libyan HCPs’ perceived barriers to CPM in the present study could be due to their lack of knowledge and training in CPM. Studies showed that a lack of knowledge and training in CPM among HCPs was reported as a significant barrier to effective CPM [19, 22]. For instance, a survey reported that lack of knowledge about CPM among approximately 61% of HCPs was one of the most frequently cited barriers to CPM [22]. Although many earlier studies showed that professional training in CPM could enhance HCPs’ knowledge and attitudes towards CPM [3, 8, 19], in the current study, Libyan HCPs with training and without training in CPM reported similar barrier scores to effective CPM. This suggests that short training in CPM was insufficient to enhance Libyan HCPs’ attitudes and knowledge about CPM. However, only 19 out of 152 Libyan HCPs in this study had training in CPM. Thus, it can be said that Libyan HCPs had a relative lack of training in CPM, which can be a further barrier to effective CPM in Libya.

Despite that, several studies have shown that work experience in cancer care settings can develop the standard of CPM services [3, 23]; in this study, both groups of HCPs who had short-term (less than 1 year) and who had long-term (greater than 1 year) work experience in cancer settings showed a similar level of barriers to CPM. This result may explain that Libyan HCPs could have inadequate knowledge and poor attitudes towards CPM due to a lack of experience in palliative care settings because palliative care service does not exist in the Libyan healthcare system [5]. A study found that HCPs who had work experience with the pain team reported adequate knowledge and positive attitudes towards CPM than those who did not [19].

However, it can be argued that direct experience in oncology or palliative care settings without professional education and training in CPM is not enough to improve HCPs’ knowledge and attitudes about CPM. This view was supported by Bernardi et al. [24], who reported that pain knowledge scores among oncology nurses were not correlated to their years of work experience. Furthermore, a survey found that HCPs who worked in clinics with academic attachments (training and education) showed notably more adequate knowledge and positive attitudes towards CPM than those in non-academic hospitals [22]. Several studies have considered the effects of educational interventions on HCPs’ attitudes and knowledge toward CPM [3, 25]. Therefore, professional education and continuous training in CPM, including opioid treatment, phobia, and addressing myths on opioid usage among HCPs [26] while further establishing policies and procedures for adopting palliative care services into the Libyan healthcare system, are needed to improve CPM in Libya.

### Strengths and Limitations

To our knowledge, this was the first survey to evaluate knowledge, attitudes, and potential barriers regarding CPM among oncology nurses and physicians in Libya. Furthermore, considering the multicenter settings in three different geographical regions of Libya (Eastern, North-western, and Western), outcomes can be better generalized to represent the sample population in question. This study has some limitations; only oncology nurses and physicians were surveyed, and other HCPs, who might prescribe opioids for CPM, such as surgeons, anaesthesiologists, and general practitioners (GP), were excluded. The use of convenience sampling could be another limitation, as it can lead to sampling bias and limit the generalizability of the findings [16]. Among the limitation of the current study is that the use of BQ-II with HCPs has been found only in six studies [11, 12, 14, 27–29], and the psychometric properties and findings of the BQ-II are not known when used for HCPs. It was originally designed as a self-assessment instrument for patient-related barriers to CPM [7]. Thus, BQ-II is a reliable and valid measure of patient-related barriers to CPM. However, BQ-II has been valid and used to measure HCP-related barriers to CPM in six previous studies [11, 12, 14, 27–29], including the Arabic version [11, 12, 14]. Another limitation is that the analyses are intended to be exploratory and hypothesis-generating; therefore, no correction has been made, and the results are interpreted with caution.

### Implications for Practice and/or Research

To enhance education concerning CPM in Libya, practical actions are needed: (1) teaching Libyan HCPs about CPM to overcome myths/misconceptions about opioids and cancer pain, especially concerning side effects of opioids, poor tolerance, and drug addiction. (2) Pain management and palliative care should be incorporated into the curriculum of Schools of Medicine and Nursing in Libya to improve education about CPM. (3) The results can be used as key issues in education and training programs to improve Libyan HCPs’ attitudes and knowledge about CPM. (4) According to the results from this study, Libyan HCPs need professional education and continuing training in CPM to reduce HCPs’ attitudinal barriers to CPM in the practice and to improve CPM knowledge among Libyan HCPs.

For research implications, based on the limitations of this study, further research with random sampling or mixed-methods study, involving other HCPs, such as surgeons, anaesthesiologists, and general practitioners (GP), is required to enhance the undersanding of potential barriers to effective CPM. Furthermore, a reseach involving additional psychometric testing of the Arabic version of BQ-II is required for reliablity and validity of the instrument.

## Conclusion

This survey showed that Libyan oncology HCPs’ perceived barriers to CPM related to lack of knowledge and poor attitudes toward CPM. Based on the results of this study, the authors recommend developing strategies, including professional education and continuous training in CPM, addressing phobia and myths about opioid usage, and improved education on the benefits and complications of using opioids for CPM among HCPs involved in the care of cancer patients.

## Supplementary Information

Below is the link to the electronic supplementary material.

Supplementary file1 (DOCX 62 KB)

## Data Availability

Data available on request from the authors: the data that support the findings of this study are available from the corresponding author (SM), upon reasonable request.
