# Supplementary material for: Attitudes, Knowledge, and Perceived Barriers Towards Cancer Pain Management Among Healthcare Professionals in Libya: a National Multicenter Survey
Source: J Cancer Educ. 2022 Jun 2;38(3):789–97. doi: 10.1007/s13187-022-02185-5 (PMC10235140; doi:10.1007/s13187-022-02185-5)
Supplement: Supplementary file 1 — Supplementary file1 (DOCX 62 KB) [file 13187_2022_2185_MOESM1_ESM.docx]

Supplementary materials

**Attitudes, knowledge, and perceived barriers towards cancer pain management among healthcare professionals in Libya: A national multicentre survey**

--------------------------------------------------------------------------------------------------------------------------

**Appendix 1** **Information sheet for Participants**

**Research title:** Attitudes, knowledge, and perceived barriers towards cancer pain management among healthcare professionals in Libya: A national multicentre survey

You are kindly invited to take part in this research study; it is crucial that you understand why the research is being done and what it involves. Therefore, please take your time to read the following information carefully before you decide to continue. Please ask if there is anything that is not clear or understandable, or if you would like further information. Take as much time as you would like to decide if you want to take part in this study or not.

1. **What is the purpose of the study?**

Recent reviews and studies reported a lack of knowledge and negative attitudes towards cancer pain management (CPM) among Health care professionals (HCPs) (oncology nurses and physicians) as one of the most common barriers to effective CPM. However, there is still no published study about HCPs’ attitudes, knowledge, and perceived barriers towards CPM in Libya. Therefore, this study evaluates HCPs’ knowledge, attitudes, and potential barriers regarding CPM in Libya.

1. **Why do we need this information?**

This information will help us to recognise the potential attitudes, knowledge, and perceived barriers about CPM among HCPs in Libya. The findings of this survey will help us in the future, in order to establish palliative care services in Libya, as well as to guide the future development of relevant educational programs for HCPs to assist cancer patients in managing their pain.

1. **Who will be involved in the research, and where will the research take place?**

The survey will be undertaken at the five different cancer national institutes and centres in Libya. The participants will be the Libyan HCPs (oncology nurses and physicians).

1. **How will the research be carried out?**

After the approval from the University of Leeds information sheets is granted. The barriers questionnaire II (BQ-II) will be distributed by the survey co-ordinators (oncology doctors; not senior or manager) to all potential participants during their break-time. The BQ-II has included a 27-item self-report questionnaire that assesses concerns about cancer pain and using pain medication for CPM. All questionnaires will take place in 5 national oncology centres in Libya. This questionnaire will take about 10 minutes in length. Simultaneously, the background information sheet for participants will be distributed before the survey, which will take about 5 minutes to complete.

1. **Do I have to take part in this study?**

The participant is anonymised, and as the participants are a volunteer, so they do not have to take part in this survey. The questionnaires cannot be withdrawn once they have been returned to the co-ordinator as they are anonymous.

1. **What will happen to me if I take part in this study?**

If you are willing to participate in the survey, you will be asked to read this information sheet and ask any questions before you sign a consent form. You will then participate in filling the questionnaire, which takes about 10 minutes.

1. **Are there any risks or benefits involved in the study?**

There will be no physical risks, as there will be no invasive procedures as a part of this study. You will not directly benefit from taking part, but the findings of the study may help to improve CPM in the future.

1. **What will happen to the research results?**

The results collected in this survey will be used in the thesis and for future publication.

1. **Will my taking part in this study be kept confidential?**

Yes, all data will be kept strictly confidential, and nobody will have access to data except the researcher and their supervisors. Any personal information obtained from participants will be stored for a period of three years following completion of the study, after which it will then be destroyed by electronic deletion and shredding as appropriate.

1. **Does this research have sufficient ethical approvals?**

Ethical approval has been sought from the School of Medicine Research Ethics Committee (The SOMREC reference is MREC 20-005; in Leeds, the UK). Besides, approval letters have been received from three national cancer institutes and centres in Libya: Tobruk Medical Centre (TMC), National Cancer Institute of Misratah (NCIM), Sabha Oncology Centre (SOC).

Thank you for taking the time to read this information sheet.

**Researcher’s contact details**

I will be always happy to clarify any things, which is not clear or giving more information regarding this study. Thus, please do not hesitate to contact me on the following details:

**Appendix 2** **Background information for participants**

Participant Number: -----------

Date: -----------------

**1. The participant is a:** (1) Nurse □ (2) Physician □

**2. Gender:** (1) Male □ (2) Female □

**3. Age in years:** ------------

**3. Check the box of the highest educational qualifications:**

(1) Secondary school □ (2) Intermediate Diplom □ (3) Undergraduate □

(4) Postgraduate □

**4. What is your current marital status?**

(1) Married □ (2) Widowed □ (3) Divorced □ (4) Not married □ (5) Engaged □

**5. Where do you live?**

(1) City □ (2) Village □ (3) Camp □

**6. Approximately what is your monthly income (LYD, convert to Pound Sterling will be done by researcher)?**

(1) No income□ (2) less than £20,000□ (3) £20,000~£39,999□

(4) £40,000~£59,999□ (5) £60,000~£79,999□ (6) £80,000~£99,999□

(7) £100,000~£149,999 □ (8) £150,000~£199,999□ (9) more than 200,000□

**7. Have you attended any training or education courses in CPM?**

(1) Yes □ (2) No □

**8. How long have you been in this job?**

(1) Six months □ (2) Less than one year □ (3) Greater than one year □

**9. Do you follow any specific guidelines for palliative care/ CPM?**

(1) Yes □ (2) No □

(3) If the answer “yes,” which one: (a) NICE’s guidelines □ (b) WHO’s guidelines □

(4) If the answer “No,” Why not ………………………………………………………………

**10. What are the current medications you prescribe/ give for CPM?**

(1) Non- Opioid: (a) Paracetamol □ (b) NSAIDs □

(2) Weak opioid: (a) codeine or dihydro-codeine□ (b) Tramadol □

(3) Strong opioid: (a) Morphine□, (b) Alfentanil□, (d) Diamorphine□, (e) Fentanyl□, (f) Oxycodone□

(4) Others: ……………………………………

Thank you for your time to complete this questionnaire

If you have any comments regarding this survey do not hesitate to contact me on the following contact details:

**Appendix 3** **Questionnaire for survey (Arabic version)**

**رقم المشارك (**ة**) :..........................**

**عزيزي المشارك \ المشاركة:**

نحن مهتمين بمعرفة وجهة نظرك ورأيك تجاه بعض الامورالمتعلقه بعلاج الألام المصاحبة للسرطان (الحواجز والمعوقات). ستلاحظ وجود اسئلة متشابهه نرجوا منك الاجابة عن جميع الاسئلة. نرجوا منك اختيار رقم من (5-0) والتي تتناسب مع مدى موافقتك على صحة كل جزء لكل من الاسئلة التاليه.

1. **من الممكن علاج الألم الناتج عن مرض السرطان**

0 1 2 3 4 5

لا أوافق أبداً أوافق بشده

1. **هناك خطرعلي المرضى للإدمان على الأدوية المسكنه للألم**

0 1 2 3 4 5

لا أوافق أبداً أوافق بشده

1. **يصعب السيطرة على الخمول الناتج عن أخد الأدوية المسكنه للألم**

0 1 2 3 4 5

لا أوافق أبداً أوافق بشده

1. **تضعف الأدوية المسكنه للألم جهاز المناعة**

0 1 2 3 4 5

لا أوافق أبداً أوافق بشده

1. **يصعب السيطره على حالة التشويش التي تنتج عن الأدوية المسكنه للألم**

0 1 2 3 4 5

لا أوافق أبداً أوافق بشده

1. **عندما يستخدم المريض الادوية المسكنه للألم فإن جسمه يتعود عليها وتصبح غير فعاله بسرعة**

0 1 2 3 4 5

لا أوافق أبداً أوافق بشده

1. **يمنع إستخدام الأدوية المسكنه للألم قدرة المريض على معرفة إذا ما كان لديه ألم جديد ام لا**

0 1 2 3 4 5

لا أوافق أبداً أوافق بشده

1. **تستطيع الأدوية المسكنه للألم علاج الألم الناتج عن مرض السرطان**

0 1 2 3 4 5

لا أوافق أبداً أوافق بشده

1. **يتعرض العديد من مرضى السرطان للادمان على الأدوية المسكنه للألم**

0 1 2 3 4 5

لا أوافق أبداً أوافق بشده

1. **لا يمكن علاج** **الغثيان الدي تسببه الأدوية المسكنه للالم**

0 1 2 3 4 5

لا أوافق أبداً أوافق بشده

1. **من المهم أن يكون المريض قويا وذلك بعدم التحدث عن ألمه**

0 1 2 3 4 5

لا أوافق أبداً أوافق بشده

1. **من المهم أن يركيز الأطباء على شفاء المرض نفسه وعدم اضاعة الوقت بعلاج الألام**

0 1 2 3 4 5

لا أوافق أبداً أوافق بشده

1. **ان إستخدام الادوية المسكنه للألم يضر الجهاز المناعي للمريض**

0 1 2 3 4 5

لا أوافق أبداً أوافق بشده

1. **ان الادوية المسكنه للألم تجعل المريض يقول ويفعل أشياء محرجه**

0 1 2 3 4 5

لا أوافق أبداً أوافق بشده

1. **إذا تناول المريض الأدوية المسكنه للألم لعلاج الم خفيف فان فاعليتها تقل في حال الألم الشديد**

0 1 2 3 4 5

لا أوافق أبداً أوافق بشده

1. **الأدوية المسكنه للألم تجعل المريض لا يعرف ما يجري داخل جسمه**

0 1 2 3 4 5

لا أوافق أبداً أوافق بشده

**17) الإمساك الذي تسببه الادوية المسكنه للألم لا يمكن تخفيفه**

0 1 2 3 4 5

لا أوافق أبداً أوافق بشده

**18 )** **اذا كان على الاطباء التعامل مع الألم فإنهم لن يركزوا على علاج المرض نفسه**

0 1 2 3 4 5

لا أوافق أبداً أوافق بشده

1. **الأدوية المسكنة ممكن تئذي الجهاز المناعي للمريض**

0 1 2 3 4 5

لا أوافق أبداً أوافق بشده

**20 )** **إنه من الأسهل للمرضى التعامل مع الألم بدل من التعامل مع الأعراض الجانبيه للأدوية المسكنه للألم**

0 1 2 3 4 5

لا أوافق أبداً أوافق بشده

**21)** **إذا المريض تناول الادوية المسكنه لللأم الان، فإنها لن تعمل بنفس الفاعليه فيما بعد**

0 1 2 3 4 5

لا أوافق أبداً أوافق بشده

**22) يمكن** **للأدوية المسكنه للألم ان تخفي التغيرات في صحة المريض**

0 1 2 3 4 5

لا أوافق أبداً أوافق بشده

**23)** **الأدوية المسكنه للألم** **إدمانيه (تسبب الادمان)**

0 1 2 3 4 5

لا أوافق أبداً أوافق بشده

1. **يمكن تخفيف** **الألام المصاحبة للسرطان** **بأستخدام الأدوية المسكنه**

0 1 2 3 4 5

لا أوافق أبداً أوافق بشده

1. **قد يجد الأطباء انه من المزعج أن يخبرهم المريض عن ألمه**

0 1 2 3 4 5

لا أوافق أبداً أوافق بشده

1. **إخبار الأطباء عن الألم قد يشتتهم عن علاج المرض نفسه**

0 1 2 3 4 5

لا أوافق أبداً أوافق بشده

1. **إذا تكلم المريض عن الألم، فإن الناس سيعتقدون انه كثير الشكوى**

0 1 2 3 4 5

لا أوافق أبداً أوافق بشده

شكرًا لمشاركتك

**Appendix 4** **Questionnaire for survey (English version)**

**Appendix 5** **Approval letter from the University**

MREC 20-005 - Attitudes, knowledge, and perceived barriers towards cancer pain management among healthcare professionals in Libya: A national multicentre survey

NB: All approvals/comments are subject to compliance with current University and UK Government advice regarding the Covid-19 pandemic.

I am pleased to inform you that the above research ethics application has been reviewed by the School of Medicine Research Ethics Committee and on behalf of the Chairs, I can confirm a favourable ethical opinion based on the documentation received at date of this email.

Please retain this email as evidence of approval in your study file.

Please notify the committee if you intend to make any amendments to the original research as submitted and approved to date. This includes recruitment methodology; all changes must receive ethical approval prior to implementation. Please see

https://leeds365.sharepoint.com/sites/ResearchandInnovationService/SitePages/Amendments.aspx or contact the Research Ethics Administrator for further information FMHUniEthics@leeds.ac.uk if required.

Ethics approval does not infer you have the right of access to any member of staff or student or documents and the premises of the University of Leeds. Nor does it imply any right of access to the premises of any other organisation, including clinical areas. The committee takes no responsibility for you gaining access to staff, students and/or premises prior to, during or following your research activities.

Please note: You are expected to keep a record of all your approved documentation, as well as documents such as sample consent forms, risk assessments and other documents relating to the study. This should be kept in your study file, which should be readily available for audit purposes. You will be given a two week notice period if your project is to be audited.

It is our policy to remind everyone that it is your responsibility to comply with Health and Safety, Data Protection and any other legal and/or professional guidelines there may be.

I hope the study goes well.

Best wishes

On behalf of SOMREC

Research Ethics Administrator

The Secretariat,

University
